# Detection and Phylogenetic Characterization of Influenza D in Swedish Cattle

**DOI:** 10.3390/v17010017

**Published:** 2024-12-26

**Authors:** Ignacio Alvarez, Fereshteh Banihashem, Annie Persson, Emma Hurri, Hyeyoung Kim, Mariette Ducatez, Erika Geijer, Jean-Francois Valarcher, Sara Hägglund, Siamak Zohari

**Affiliations:** 1 Division of Ruminant Medicine, Department of Clinical Sciences, Swedish University of Agricultural Sciences, P.O. Box 7054, 756 51 Uppsala, Sweden; 2 Department of Microbiology, Swedish Veterinary Agency, Ulls väg 2B, 751 89 Uppsala, Swedensiamak.zohari@sva.se (S.Z.); 3 Department of Clinical Sciences, Swedish University of Agricultural Sciences, 756 51 Uppsala, Sweden; 4 Department of Animal Health and Antimicrobial Strategies, Swedish Veterinary Agency, 751 89 Uppsala, Sweden; 5 Department of Epidemiology, Surveillance and Risk Assessment, Swedish Veterinary Agency, Ulls väg 2B, 751 89 Uppsala, Sweden; 6 Interactions Hôtes-Agents-Pathogènes, Ecole Vétérinaire de Toulouse (ENVT), Institut National de Recherche pour l’Agriculture, l’Alimentation et l’Environnement, 31300 Toulouse, France; 7 Gård & Djurhälsan, Kungsängens Gård, 753 23 Uppsala, Sweden

**Keywords:** influenza D virus, cattle, bovine, PCR, Sweden, co-infection, phylogeny, sequencing, reassortment

## Abstract

Increased evidence suggests that cattle are the primary host of Influenza D virus (IDV) and may contribute to respiratory disease in this species. The aim of this study was to detect and characterise IDV in the Swedish cattle population using archived respiratory samples. This retrospective study comprised a collection of a total 1763 samples collected between 1 January 2021 and 30 June 2024. The samples were screened for IDV and other respiratory pathogens using real-time reverse transcription quantitative PCR (rRT-qPCR). Fifty-one IDV-positive samples were identified, with a mean cycle threshold (Ct) value of 27 (range: 15–37). Individual samples with a Ct value of <30 for IDV RNA were further analysed by deep sequencing. Phylogenetic analysis was performed by the maximum likelihood estimation method on the whole IDV genome sequence from 16 samples. The IDV strains collected in 2021 (n = 7) belonged to the D/OK clade, whereas samples from 2023 (n = 4) and 2024 (n = 5) consisted of reassortants between the D/OK and D/660 clades, for the PB2 gene. This study reports the first detection of IDV in Swedish cattle and the circulation of D/OK and reassortant D/OK-D/660 in this population.

## 1. Introduction

In 2011, a virus similar to influenza C was identified in pigs with respiratory symptoms in the USA [1]. Due to its distinct genetic profile and lack of cross-reactivity with influenza C, it was reclassified as a new genus, now recognised as influenza D virus (IDV).

IDV can infect and transmit across several species, with cattle identified as the primary host. The presence of IDV in both healthy and sick animals suggests limited virulence in cattle [2,3]. However, metagenomic studies have implicated IDV in bovine respiratory disease (BRD) [4,5]. While experimental intranasal infections in calves have induced mild lesions in the respiratory tract, co-infections with *Mycoplasma bovis* have resulted in more severe lesions and clinical signs, suggesting a potential role of IDV as a co-pathogen in BRD [6].

Despite the limited or null virulence of IDV reported in other animal species to date, there is still reason to be concerned about the potential role of IDV in humans as a zoonotic disease. Several studies have shown the presence of IDV antibodies in human sera, with a wide range of positivity among different geographical and professional populations [7,8,9]. In Florida, a seroprevalence of 97% was reported among cattle workers, compared to 18% among individuals without cattle contact, while in Italy, 46% of serum samples were IDV antibody-positive by HI assay in 2014 [7,9]. In addition to serological tests, IDV has been detected once in a bioaerosol from a hospital and in an airport, and in vitro studies demonstrate the ability of IDV to replicate in human respiratory epithelial cells and also to infect and transmit in ferrets, mice, and guinea pigs, which are common models used to study influenza infection in humans [1,10,11,12,13,14].

The virulence potential of influenza viruses is strongly linked to genetic characteristics allowing spreading between different hosts. Since their discovery, IDV strains have been divided based on the hemagglutinin-esterase fusion (HEF) gene into four major lineages. In Europe, two major lineages have been detected so far, D/OK and D/660 [15]. In 2022, Gaudino et al. demonstrated that the HEF glycoprotein of IDV has a significantly higher nucleotide substitution rate compared to the HEF of human influenza C virus, suggesting the possibility for reassortment and the emergence of new strains [15]. Genetic reassortant patterns in IDV, comprising gene segments from both D/OK and D/660, have previously been observed in Italy, USA, Canada, and France [16,17,18,19].

Previously in Sweden, the circulation of IDV was indicated by the detection of IDV-specific antibodies in 282 out of 799 (35%) bulk milk samples from Swedish dairy farms, but the IDV strains circulating in the cattle population were not detected or phylogenetically characterised [20]. This study aimed to screen respiratory samples collected from cattle with respiratory signs by IDV real-time reverse transcription quantitative PCR (RT-qPCR), to determine the presence of IDV in sampled animals and to perform a phylogenetic characterisation to improve the understanding of the evolutionary dynamics of IDV in Sweden.

## 2. Materials and Methods

A total of 1763 respiratory samples collected from bovine clinical cases exhibiting respiratory signs were retrospectively analysed for the presence of IDV. These samples, submitted to the Swedish Veterinary Agency between 1 January 2021, and 30 June 2024, consisted of lung tissue and individual or pooled nasal swabs, with each pool containing samples from up to five animals. Samples were collected in 248, 258, 248, and 239 herds in 2021, 2022, 2023, and 2024, respectively. The farms were geographically spread through the whole country and were located in 172 municipalities from the Övertorneå Municipality (66°23′17″ N 23°39′13″ E) in the North to the Trelleborg Municipality (55°22′ N 13°10′ E) in the Southern part of the country. The samples were initially screened for a range of the most common respiratory pathogens, including bovine coronavirus (BCoV), bovine respiratory syncytial virus (BRSV), bovine parainfluenza virus type 3, *Mycoplasma bovis*, *Pasteurella multocida*, *Histophilus somni*, and *Mannheimia haemolytica*, as part of routine diagnostics and veterinary care investigation and stored at −80 °C until tested for IDV. If IDV was detected in any pooled samples, a confirmatory PCR test was performed on individual samples from that pool. This step not only reconfirmed the presence of IDV, but also determined the extent of positive animals among that pool. Positive samples for IDV were then used for phylogenetic analysis.

RNA was extracted using the IndiMag Pathogen Kit (INDICAL Bioscience, Leipzig, Germany). For each extraction, 200 μL of the sample and 20 μL of proteinase K were transferred to a Deep Well-96 plate, followed by the addition of 500 μL of lysate mix, prepared according to the manufacturer’s instructions. The extraction process was carried out using a Maelstrom-9600 (TANBead, Taoyuan City, Taiwan) extraction robot. The presence of IDV was detected using RT-qPCR, targeting the NP gene of IDV, as previously described [21]. Positive samples were retested in triplicate using a second RT-qPCR assay targeting the polymerase gene of influenza D viruses to confirm the results [1]. IDV-RNA-positive samples with Ct values <30 were selected for whole-genome sequencing. These samples underwent metagenomic next-generation sequencing using the Illumina MiSeq instrument (Illumina Inc., San Diego, CA, USA). Library construction was performed with the NEXTERA-XT kit (Illumina Inc., San Diego, CA, USA), according to the manufacturer’s instructions. The quality of the libraries was assessed using the Agilent 2100 Bioanalyzer (Agilent Technologies, Santa Clara, CA, USA). Sequencing was conducted on a MiSeq instrument using a MiSeq Reagent Kit v3 in a 600-cycle paired-end run. The generated data were analysed with CLC Genomics Workbench v21 (CLC bio, Aarhus, Denmark).

Phylogenetic trees were constructed for each gene segment using relevant nucleotide sequences from GenBank. Blast homology searches were performed to retrieve the top 100 homologous sequences for the sequenced gene segments. Phylogenetic analysis was conducted using the maximum likelihood method implemented in MEGA7: Molecular Evolutionary Genetics Analysis version 7.0.26. The robustness of the ML trees was evaluated by bootstrap analysis with 2000 replicates.

Choropleth maps showing Sweden with the positive municipalities and regions were created by using the R version 4.4.1.

## 3. Results

**Figure 1:**
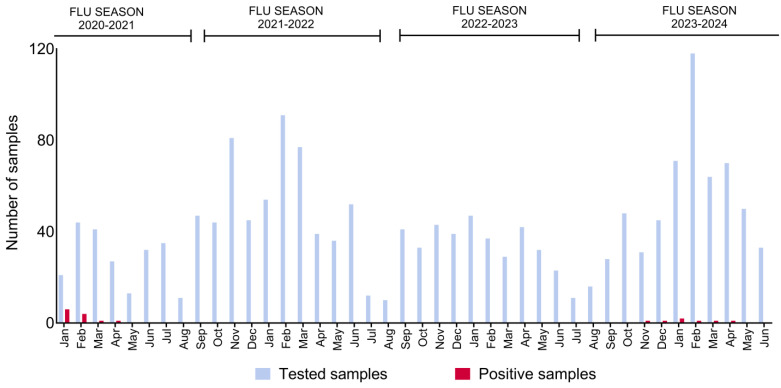
Number of tested and positive samples from 2021 to 2024, categorised by influenza seasons (October–September). Positive samples were detected during the 2020–2021 and 2023–2024 seasons.

A total of 51 animals were positive for IDV. Of these, 36 positive samples were identified during the 2020–2021 influenza season (1st of October–30th of September), while the remaining 15 were detected during the 2023–2024 season (Figure 1). The mean Ct value for IDV-positive samples was 27, with a range from 15 to 37.

Positive samples were collected from 14 different farms, 9 of which were fattening farms (Table 1). Regarding the age distribution, 38/51 (75%) positive samples were from calves younger than 16 weeks (Table 1).

Among the 51 samples that tested positive for IDV, only one animal was likely single infected with IDV, while the remaining 50 showed co-infections with at least two other pathogens from the respiratory panel. Specifically, *P. multocida* was the most common bacteria, detected in 45 IDV-positive samples (88%), whereas BCoV was the most common virus, detected in 31 IDV-positive samples (60%, Table S1). Regarding the simultaneous detection of several viruses, nine samples were positive for IDV, BCoV, and PIV-3, eight samples were positive for IDV, BCoV, and BRSV, and six samples were positive for IDV, BRSV, and PIV-3.

**Figure 2:**
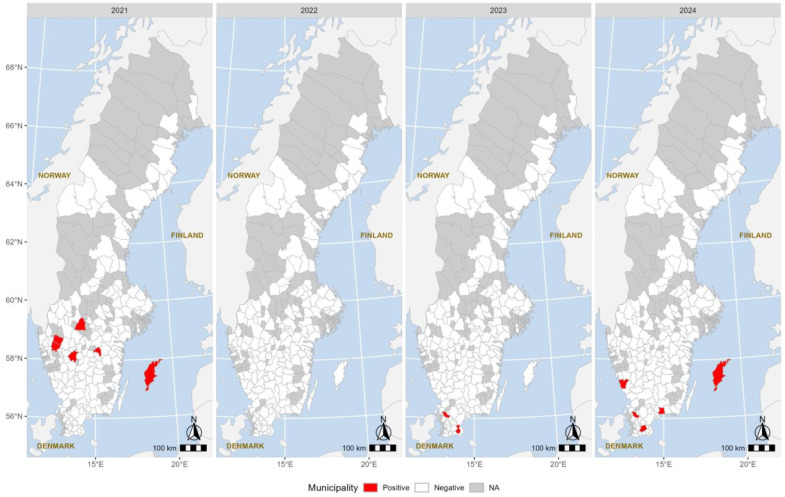
Choropleth maps showing municipalities with positive samples (in red) from 2021 to 2024.

In 2021, positive samples were detected in six municipalities: Mjölby, Falköping, Mellerud, Vänersborg, Kristinehamn, and Gotland. In 2023, Tomelilla and Klippan were the only two municipalities with positive samples. However, in 2024, the number increased again, with positive samples detected in four different municipalities: Gotland, Varberg, Klippan, and Sjöbo (Figure 2).

**Figure 3:**
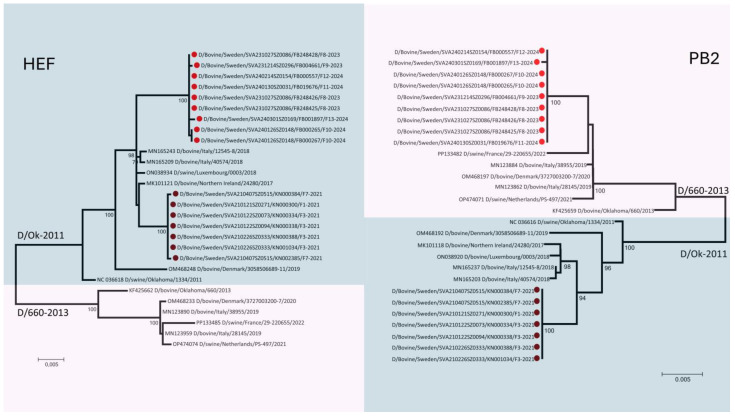
Phylogenetic trees of Influenza D virus collected in Sweden. The trees illustrate the relationships of the gene segments encoding the HEF and PB2 proteins with reference strains from the D/OK and D/660 lineages. Strains collected analysed based on HEF protein analysis clustered within the D/OK lineage. However, samples collected in 2023 and 2024 showed reassortment, with genetic material from both the D/OK and D/660 lineages, specifically with gene segment 1 clustering within the D/660 lineage.

Phylogenetic analysis of the seven gene segments of IDV revealed distinct patterns. Strains collected in 2021 (n = 7) clustered within the D/OK lineage. In contrast, strains collected in 2023–2024 (n = 9) were reassortants between the D/OK and D/660 lineages. Specifically, gene segment 1, which encodes the PB2 protein, clustered within the D/660 lineage (Figure 3), while the rest of the gene segments remained within the D/OK gene pool (Figure S1).

## 4. Discussion

In this study, we confirmed the presence of IDV by detecting the virus genome in samples from animals with respiratory disease and identified two genetic variants of influenza D viruses among cattle in Sweden. The viruses circulating in 2021 had their complete genome constructed of segments of the D/OK lineage gene pool, while segment 1 (Polymerase Basic 2-PB2 gene) of the detected viruses in the 2023–2024 season belonged to the D/660 lineage gene pool, representing a novel genotype and reassortants between the D/OK and D/660 lineages. The low detection rate suggests that IDV is not a major health concern in Swedish cattle production. Regarding age and based on our results, it seems that IDV follows the same pattern as other respiratory pathogens, being predominantly detected in calves younger than 4 months. This coincides with the time when maternal antibodies have decreased and calves are exposed to stressors [22]. The higher detection rate of positive calves in fattening farms may be linked to common conditions in this type of production, such as the mixing of calves from different farms, transportation stress, changes in diet, new housing environment, and high animal density.

Only 1 out of 51 IDV-positive animals was found to be single infected with IDV, whereas the remaining 50 were positive for at least two other pathogens from the respiratory panel. Several of the detected bacteria are part of the normal nasal flora and may not contribute to clinical signs. This underscores the challenges in distinguishing between commensal presence and pathogenic impact and highlights the limitation in the use of the molecular detection of pathogens in nasal secretions for diagnostics. Several studies have identified IDV in nasal swabs of asymptomatic animals, suggesting that subclinical infections are common. While much research on BRD focuses on interactions between viruses and bacteria, our findings indicate that co-infections involving multiple established respiratory viruses are also common. This aspect of BRD has been relatively understudied, and our results emphasise the need for further investigation into the implications of such viral interactions.

While a more extended study period is needed to assess the possibility of a cyclic or “wave-like” pattern, the detection of positive animals during the colder months of 2021 and 2023/2024 suggests some degree of seasonality or annual occurrence, similarly to what has been observed in several influenza studies, including those on IDV [7,23,24,25]. In this study, it is challenging to distinguish between the effects of the winter season on immune status and the increased presence of the virus. During winter, when temperatures drop and daylight is limited, especially in a country like Sweden, several factors could contribute to the higher detection rates. The use of indoor housing systems, more common in colder seasons, likely increases viral transmission due to closer animal proximity, reduced ventilation, and increased viral survival in the environment [26].

The discrepancy between the prevalence of IDV antibodies in the previous serological study and the limited detection of the pathogen by RT-qPCR in the present study may be due to the use of different approaches (indirect vs. direct virus detection), different sample types (bulk milk vs. respiratory samples), and differences in the study population (cows vs. calves). Bulk milk reflects the antibody status of the adult cow population, the group with the longest pathogen exposure on a dairy farm. Although the duration of antibody responses against IDV is not well defined, it is reasonable to assume that IDV-specific antibodies can remain detectable for several years, as was demonstrated for IAV virus in humans and BRSV in cattle [27,28,29]. In addition, limited information is available regarding the persistence or clearance of IDV in different tissues and samples. Based on three studies with experimental infections, the clinical signs of IDV are mild and peak between 5 and 8 days post-infection (dpi) [6,30,31]. In one of these studies, Ferguson et al. demonstrated that at 4 dpi, IDV RNA was present in both the upper and lower respiratory tracts of infected calves, but by 6 dpi, it was only detected in nasal tissue [31]. Moreover, Lion et al. reported that five out of five animals infected with IDV tested positive in nasal swabs at 8 dpi, but only two out of those five were positive at 10 dpi, and only one was at 12 dpi [30]. These results suggest that the detection window for IDV in the respiratory tract is very short. It is important to note that these studies involved experimental infections, where the viral load and the direct induction of the infection in the upper respiratory tract were probably different than under natural field conditions. The inability to detect mild clinical signs, combined with delays in sampling until more severe clinical signs, likely due to secondary infections, could explain the limited detection of animals infected with IDV. The detection of positive samples in 2021, followed by their re-emergence in 2023–2024, might serve as an example of the infection–immunity–reinfection model that can explain the cyclic pattern of IDV. After an IDV outbreak, a significant portion of the population likely develops a partial and temporary immunity, which reduces the viral circulation in the following years. As this immunity declines or new susceptible individuals are introduced into the population, the risk of viral re-emergence increases, facilitating the viral return. Sweden is characterised by a lower cattle density and higher biosecurity standards compared to other countries, which help to limit and prevent the introduction and spread of various pathogens [32].

Monitoring the genetic characteristics of IDV is crucial for tracking viral variation and understanding its impact on the clinical expression of infections, as well as tissue and animal species tropism. Phylogenetic analysis of the sequenced samples revealed that IDV strains collected in 2021 belonged to the D/OK lineage, whereas the sequenced samples from 2023 consisted of reassortants between the D/OK and D/660 lineages. Although a limited number of strains were identified, this suggests an ongoing genetic reassortment and evolution of the virus in the Swedish cattle population. Similar results have been observed in Denmark, where a shift in the circulating IDV lineage from D/OK to D/660 has been detected in recent years [33]. This shift is particularly significant when discussing influenza viruses because, in the case of IDV, it has been shown that the D/OK and D/660 lineages do not generate a fully cross-reactive immune response [16,34]. Although it is difficult to predict the exact impact of the PB2 gene reassortment, this could significantly influence the behaviour and epidemiology of the virus, potentially altering its replication and hence its virulence and transmission dynamics [35,36].

Given the current understanding of the role of IDV in bovine respiratory disease, it would be recommended to include IDV in routine diagnostic respiratory panels. This would provide opportunities to follow the prevalence and virulence, gain a more comprehensive insight into the respiratory disease complex of cattle, and improve disease management strategies in the field.

In conclusion, although IDV currently appears to have a limited impact on cattle health in Sweden, its potential role as a co-pathogen and the ongoing genetic evolution highlights the need for continued surveillance.

## Figures and Tables

**Table 1:** Laboratory results and sample information, including farm production type, sample type, and age of sampled calves. F = fattening farm, S = suckler cow, and D = dairy farm n.d = no data. NS = Nasal swabs and Lung= lung tissue. W = Week, Y = year. The results are presented as the Pos (+) and Neg (−) of the corresponding RT-qPCR for IDV.

Farm ID	Production Type	Sample Type	Age Group	Number of Tested Animals	Number of IDV-Positive Animals
1	F	NS	3–6 w	4	4
2	S	NS	1 y	4	4
3	F	NS	12–14 w	4	4
		NS	3–5 w	3	3
		NS	8–28 w	3	3
		NS	4 w	4	4
		NS	8 w	3	3
		NS	4 w	3	3
4	F	NS	8–12 w	3	1
5	n.d	NS	n.d	1	1
6	F	NS	28 w	1	1
7	F	NS	n.d	4	2
8	F	NS	4–14 w	4	4
9	F and S	Ns	49 w	1	1
10	D	NS	2–6 w	4	4
11	n.d	NS	n.d	1	1
12	F	NS	4–14 w	3	3
13	n.d	Lung	4 y	1	1
14	F	NS	8–16 w	4	4

## Data Availability

The sequences are available at www.ncbi.nlm.nih.gov/genbank (accessed on 1 December 2024), and the accession numbers are presented in Table S2.

## Supplementary Materials

The following supporting information can be downloaded at https://www.mdpi.com/article/10.3390/v17010017/s1, Table S1. Results of the laboratory analyses. Samples of nasal swabs (nasal swabs-NS, pooled nasal swabs-pNS-each pool containing three to five individual samples) and lung tissue. The production type is F = Fattening farm, S = Suckler cow and D = Dairy farm. Positive results of pooled and individual samples are presented as the Pos/Neg of the corresponding PCR; BCoV = Bovine Coronavirus, BRSV = Bovine Respiratory Syncitia virus, PIV-3 = Bovine Parainfluenza 3, M.Bovis = Mycoplasma Bovis, P. multocida = Pasturella multocida, M. haem = Mannheimia haemolytica, H.somnia = Histophilus somni, IDV = Influenza D virus, Pos. = positive qPCR result, Neg. = Negative qPCR. Table S2. Accession Number for the Sequenced Strain. Figure S1. Phylogenetic trees illustrating the relationships of the gene segments encoding the PB1, P3, NP, P42, and NS proteins with reference strains from the D/OK and D/660 lineages. Figure S2. Philogenetic trees illustrate the relationships of the gene segments encoding the HEF and PB2 proteins with reference strains from the D/OK and D/660 lineages.

## References

[1] Hause B.M., Ducatez M., Collin E.A., Ran Z., Liu R., Sheng Z., Armien A., Kaplan B., Chakravarty S., Hoppe A.D. (2013). Isolation of a novel swine influenza virus from Oklahoma in 2011 which is distantly related to human influenza C viruses. PLoS Pathog..

[2] Mekata H., Yamamoto M., Hamabe S., Tanaka H., Omatsu T., Mizutani T., Hause B.M., Okabayashi T. (2018). Molecular epidemiological survey and phylogenetic analysis of bovine influenza D virus in Japan. Transbound. Emerg. Dis..

[3] Wan X.F., Ferguson L., Oliva J., Rubrum A., Eckard L., Zhang X.J., Woolums A.R., Lion A., Meyer G., Murakami S. (2020). Limited Cross-Protection Provided by Prior Infection Contributes to High Prevalence of Influenza D Viruses in Cattle. J. Virol..

[4] Mitra N., Cernicchiaro N., Torres S., Li F., Hause B.M. (2016). Metagenomic characterization of the virome associated with bovine respiratory disease in feedlot cattle identified novel viruses and suggests an etiologic role for influenza D virus. J. Gen. Virol..

[5] Ng T.F., Kondov N.O., Deng X., Van Eenennaam A., Neibergs H.L., Delwart E. (2015). A metagenomics and case-control study to identify viruses associated with bovine respiratory disease. J. Virol..

[6] Salem E., Hagglund S., Cassard H., Corre T., Naslund K., Foret C., Gauthier D., Pinard A., Delverdier M., Zohari S. (2019). Pathogenesis, Host Innate Immune Response, and Aerosol Transmission of Influenza D Virus in Cattle. J. Virol..

[7] Trombetta C.M., Marchi S., Manini I., Kistner O., Li F., Piu P., Manenti A., Biuso F., Sreenivasan C., Druce J. (2020). Influenza D Virus: Serological Evidence in the Italian Population from 2005 to 2017. Viruses.

[8] Trombetta C.M., Montomoli E., Di Bartolo I., Ostanello F., Chiapponi C., Marchi S. (2022). Detection of antibodies against influenza D virus in swine veterinarians in Italy in 2004. J. Med. Virol..

[9] White S.K., Ma W., McDaniel C.J., Gray G.C., Lednicky J.A. (2016). Serologic evidence of exposure to influenza D virus among persons with occupational contact with cattle. J. Clin. Virol..

[10] Bailey E.S., Choi J.Y., Zemke J., Yondon M., Gray G.C. (2018). Molecular surveillance of respiratory viruses with bioaerosol sampling in an airport. Trop. Dis. Travel. Med. Vaccines.

[11] Choi J.Y., Zemke J., Philo S.E., Bailey E.S., Yondon M., Gray G.C. (2018). Aerosol Sampling in a Hospital Emergency Room Setting: A Complementary Surveillance Method for the Detection of Respiratory Viruses. Front. Public Health.

[12] Oliva J., Mettier J., Sedano L., Delverdier M., Bourges-Abella N., Hause B., Loupias J., Pardo I., Bleuart C., Bordignon P.J. (2020). Murine Model for the Study of Influenza D Virus. J. Virol..

[13] Sreenivasan C., Thomas M., Sheng Z.Z., Hause B.M., Collin E.A., Knudsen D.E.B., Pillatzki A., Nelson E., Wang D., Kaushik R.S. (2015). Replication and Transmission of the Novel Bovine Influenza D Virus in a Guinea Pig Model. J. Virol..

[14] Holwerda M., Kelly J., Laloli L., Sturmer I., Portmann J., Stalder H., Dijkman R. (2019). Determining the Replication Kinetics and Cellular Tropism of Influenza D Virus on Primary Well-Differentiated Human Airway Epithelial Cells. Viruses.

[15] Gaudino M., Chiapponi C., Moreno A., Zohari S., O’Donovan T., Quinless E., Sausy A., Oliva J., Salem E., Fusade-Boyer M. (2022). Evolutionary and temporal dynamics of emerging influenza D virus in Europe (2009-22). Virus Evol..

[16] Collin E.A., Sheng Z.Z., Lang Y.K., Ma W.J., Hause B., Li F. (2015). Cocirculation of Two Distinct Genetic and Antigenic Lineages of Proposed Influenza D Virus in Cattle. J. Virol..

[17] Chiapponi C., Faccini S., Fusaro A., Moreno A., Prosperi A., Merenda M., Baioni L., Gabbi V., Rosignoli C., Alborali G.L. (2019). Detection of a New Genetic Cluster of Influenza D Virus in Italian Cattle. Viruses.

[18] Gorin S., Richard G., Herve S., Eveno E., Blanchard Y., Jardin A., Rose N., Simon G. (2024). Characterization of Influenza D Virus Reassortant Strain in Swine from Mixed Pig and Beef Farm, France. Emerg. Infect. Dis..

[19] Saegerman C., Gaudino M., Savard C., Broes A., Ariel O., Meyer G., Ducatez M.F. (2022). Influenza D virus in respiratory disease in Canadian, province of Quebec, cattle: Relative importance and evidence of new reassortment between different clades. Transbound. Emerg. Dis..

[20] Alvarez I., Hagglund S., Naslund K., Eriksson A., Ahlgren E., Ohlson A., Ducatez M.F., Meyer G., Valarcher J.F., Zohari S. (2023). Detection of Influenza D-Specific Antibodies in Bulk Tank Milk from Swedish Dairy Farms. Viruses.

[21] Henritzi D., Hoffmann B., Wacheck S., Pesch S., Herrler G., Beer M., Harder T.C. (2019). A newly developed tetraplex real-time RT-PCR for simultaneous screening of influenza virus types A, B, C and D. Influenza Other Resp..

[22] Barrington G.M., Parish S.M. (2001). Bovine neonatal immunology. Vet. Clin. N. Am. Food Anim. Pract..

[23] Yu J.S., Li F., Wang D. (2021). The first decade of research advances in influenza D virus. J. Gen. Virol..

[24] He D., Lui R., Wang L., Tse C.K., Yang L., Stone L. (2015). Global Spatio-temporal Patterns of Influenza in the Post-pandemic Era. Sci. Rep..

[25] Horimoto T., Hiono T., Mekata H., Odagiri T., Lei Z., Kobayashi T., Norimine J., Inoshima Y., Hikono H., Murakami K. (2016). Nationwide Distribution of Bovine Influenza D Virus Infection in Japan. PLoS ONE.

[26] Welliver R.C. (2007). Temperature, humidity, and ultraviolet B radiation predict community respiratory syncytial virus activity. Pediatr. Infect. Dis. J..

[27] Chen J., Zhu H., Horby P.W., Wang Q., Zhou J., Jiang H., Liu L., Zhang T., Zhang Y., Chen X. (2020). Specificity, kinetics and longevity of antibody responses to avian influenza A(H7N9) virus infection in humans. J. Infect..

[28] Hagglund S., Naslund K., Svensson A., Lefverman C., Enul H., Pascal L., Siltenius J., Holzhauer M., Delabouglise A., Osterberg J. (2022). Longitudinal study of the immune response and memory following natural bovine respiratory syncytial virus infections in cattle of different age. PLoS ONE.

[29] Krammer F. (2019). The human antibody response to influenza A virus infection and vaccination. Nat. Rev. Immunol..

[30] Lion A., Secula A., Rancon C., Boulesteix O., Pinard A., Deslis A., Hagglund S., Salem E., Cassard H., Naslund K. (2021). Enhanced Pathogenesis Caused by Influenza D Virus and Mycoplasma bovis Coinfection in Calves: A Disease Severity Linked with Overexpression of IFN-gamma as a Key Player of the Enhanced Innate Immune Response in Lungs. Microbiol. Spectr..

[31] Ferguson L., Olivier A.K., Genova S., Epperson W.B., Smith D.R., Schneider L., Barton K., McCuan K., Webby R.J., Wan X.F. (2016). Pathogenesis of Influenza D Virus in Cattle. J. Virol..

[32] Noremark M., Frossling J., Lewerin S.S. (2010). Application of routines that contribute to on-farm biosecurity as reported by Swedish livestock farmers. Transbound. Emerg. Dis..

[33] Goecke N.B., Liang Y., Otten N.D., Hjulsager C.K., Larsen L.E. (2022). Characterization of Influenza D Virus in Danish Calves. Viruses.

[34] Wan Y.M., Kang G.B., Sreenivasan C., Daharsh L., Zhang J.F., Fan W.J., Wang D., Moriyama H., Li F., Li Q.S. (2018). A DNA Vaccine Expressing Consensus Hemagglutinin-Esterase Fusion Protein Protected Guinea Pigs from Infection by Two Lineages of Influenza D Virus. J. Virol..

[35] Chauhan R.P., Gordon M.L. (2022). An overview of influenza A virus genes, protein functions, and replication cycle highlighting important updates. Virus Genes.

[36] Wang Y., Sun W., Wang Z., Zhao M., Zhang X., Kong Y., Wang X., Feng N., Wang T., Yan F. (2021). Amino acid sites related to the PB2 subunits of IDV affect polymerase activity. Virol. J..

