# Supplementary figures and images for: Detection and Phylogenetic Characterization of Influenza D in Swedish Cattle
Source: Viruses. 2024 Dec 26;17(1):17. doi: 10.3390/v17010017 (PMC11768518; doi:10.3390/v17010017)

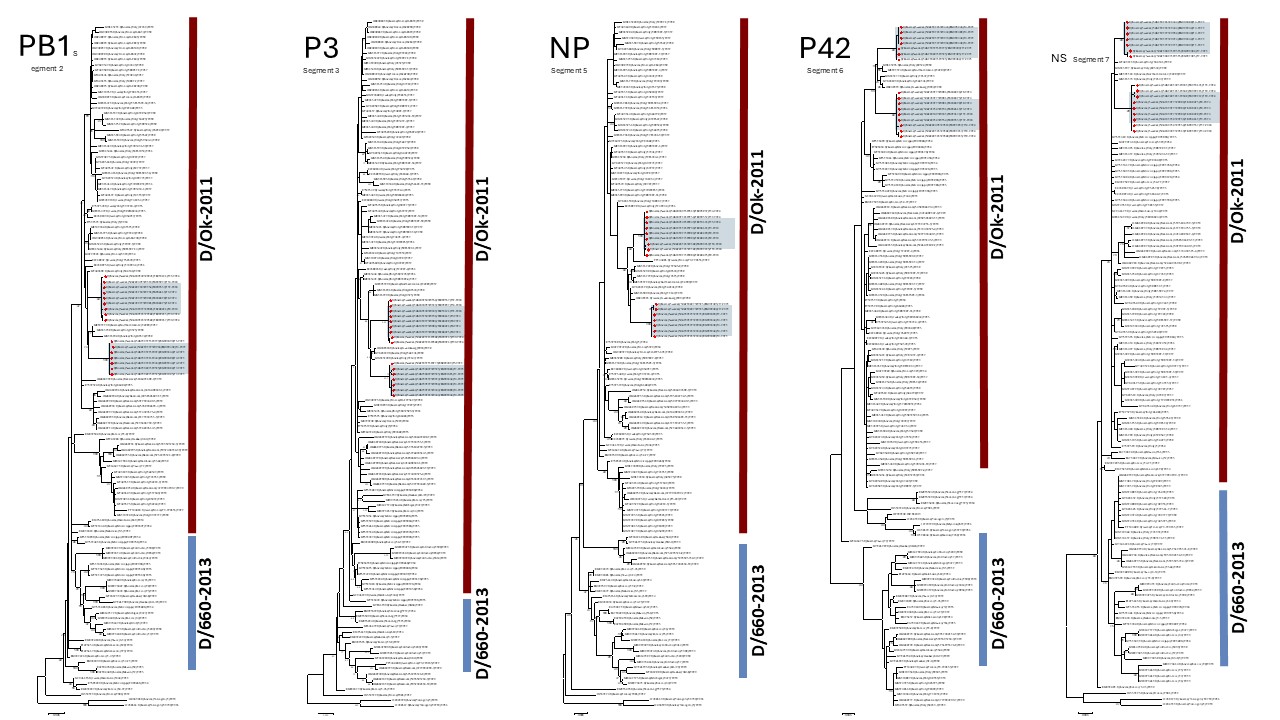

Supplement: Supplementary file 1 [file viruses-17-00017-s001.zip › viruses-3355050-supplementary/Figure S1. Phylogenetic tree .jpg]

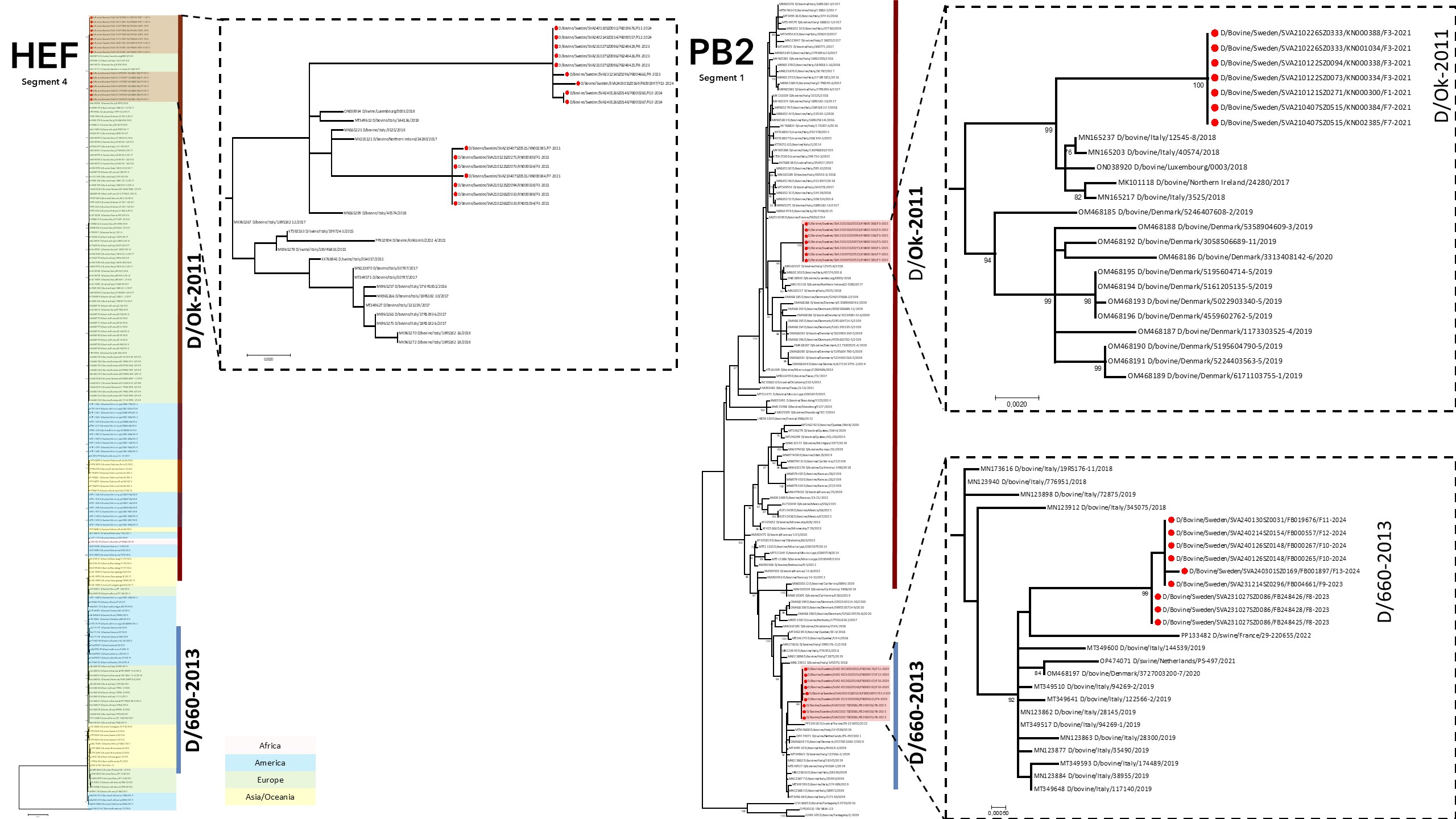

Supplement: Supplementary file 1 [file viruses-17-00017-s001.zip › viruses-3355050-supplementary/Figure S2. Phylogenetic tree.jpg]
